# 3a,8a-Dihy­droxy-1,3,3a,8a-tetra­hydro­indeno­[1,2-*d*]imidazole-2,8-dione

**DOI:** 10.1107/S1600536811019039

**Published:** 2011-05-25

**Authors:** Raza Murad Ghalib, Rokiah Hashim, Sayed Hasan Mehdi, Ching Kheng Quah, Hoong-Kun Fun

**Affiliations:** a School of Industrial Technology, Universiti Sains Malaysia, 11800 USM, Penang, Malaysia; b X-ray Crystallography Unit, School of Physics, Universiti Sains Malaysia, 11800 USM, Penang, Malaysia

## Abstract

In the title mol­ecule, C_10_H_8_N_2_O_4_, the imidazolidine ring adopts a twisted conformation. In the crystal, the mol­ecules are linked *via* a pair of bifurcated inter­molecular O—H⋯O hydrogen bonds, forming an inversion dimer. The dimers are further linked *via* N—H⋯O hydrogen bonds into a tape along the *b* axis.

## Related literature

For general background to and the properties of ninhydrinurea derivatives, see: Caputo *et al.* (1987 ▶); Kaupp *et al.* (2002 ▶); Sarra & Stephani (2000 ▶). For standard bond-length data, see: Allen *et al.* (1987 ▶). For ring conformations, see: Cremer & Pople (1975 ▶).
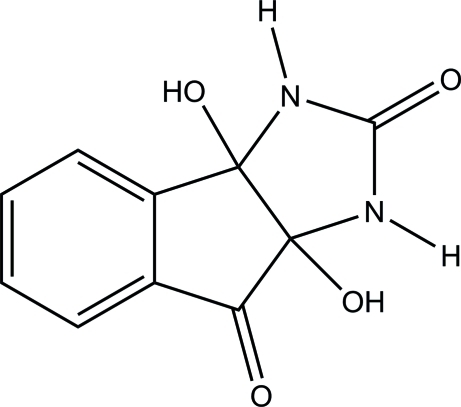


## Experimental

### 

#### Crystal data

C_10_H_8_N_2_O_4_
*M* *_r_* = 220.18
Triclinic, 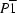

*a* = 6.7914 (2) Å
*b* = 7.3201 (2) Å
*c* = 9.5006 (3) Å
α = 94.258 (1)°
β = 102.773 (1)°
γ = 93.725 (1)°
*V* = 457.74 (2) Å^3^
*Z* = 2
Mo *K*α radiation
μ = 0.13 mm^−1^
*T* = 296 K
0.39 × 0.15 × 0.05 mm

#### Data collection

Bruker SMART APEXII CCD area-detector diffractometer
Absorption correction: multi-scan (*SADABS*; Bruker, 2009 ▶) *T*_min_ = 0.952, *T*_max_ = 0.994
6684 measured reflections
2081 independent reflections
1634 reflections with *I* > 2σ(*I*)
*R*_int_ = 0.022

#### Refinement

*R*[*F* ^2^ > 2σ(*F* ^2^)] = 0.040
*wR*(*F* ^2^) = 0.110
*S* = 1.05
2081 reflections
177 parameters
H atoms treated by a mixture of independent and constrained refinement
Δρ_max_ = 0.25 e Å^−3^
Δρ_min_ = −0.23 e Å^−3^

### 

Data collection: *APEX2* (Bruker, 2009 ▶); cell refinement: *SAINT* (Bruker, 2009 ▶); data reduction: *SAINT*; program(s) used to solve structure: *SHELXTL* (Sheldrick, 2008 ▶); program(s) used to refine structure: *SHELXTL*; molecular graphics: *SHELXTL*; software used to prepare material for publication: *SHELXTL* and *PLATON* (Spek, 2009 ▶).

## Supplementary Material

Crystal structure: contains datablocks global, I. DOI: 10.1107/S1600536811019039/is2712sup1.cif

Structure factors: contains datablocks I. DOI: 10.1107/S1600536811019039/is2712Isup2.hkl

Supplementary material file. DOI: 10.1107/S1600536811019039/is2712Isup3.cml

Additional supplementary materials:  crystallographic information; 3D view; checkCIF report

## Figures and Tables

**Table 1:** Hydrogen-bond geometry (Å, °)

*D*—H⋯*A*	*D*—H	H⋯*A*	*D*⋯*A*	*D*—H⋯*A*
O3—H1*O*3⋯O1^i^	0.89 (3)	2.02 (3)	2.8653 (17)	158 (2)
O2—H1*O*2⋯O1^i^	0.95 (3)	1.89 (3)	2.8103 (17)	163 (2)
N2—H1*N*2⋯O4^ii^	0.83 (2)	2.454 (19)	3.1282 (19)	139.3 (18)
N1—H1*N*1⋯O4^iii^	0.86 (2)	2.06 (2)	2.8841 (18)	159.4 (19)

Symmetry codes: (i) 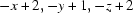; (ii) 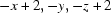; (iii) 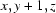.

**Fig. 1.**
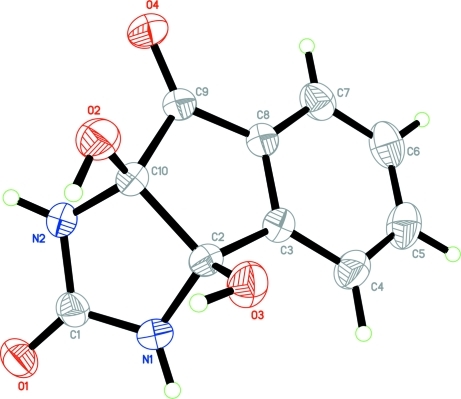
The molecular structure of the title compound, showing 50% probability displacement ellipsoids for non-H atoms.

**Fig. 2.**
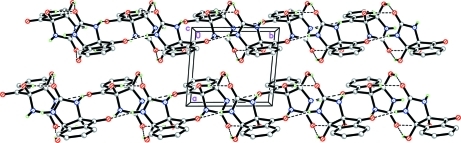
The crystal structure of the title compound, viewed along the c axis. H atoms not involved in hydrogen bonds (dashed lines) have been omitted for clarity.

## supplementary crystallographic information

### Comment

The title compound ninhydrinurea (Caputo *et al.*, 1987; Kaupp *et al.*, 2002; Sarra & Stephani, 2000) has been synthesized by a new route.

In the title compound, Fig. 1, the imidazolidine ring (N1/N2/C1/C2/C10) is twisted about the N2—C10 bond with puckering parameters (Cremer & Pople, 1975) Q = 0.1107 (16) Å and Θ =  271.8 (8)° and its least-squares plane makes a dihedral angle of 62.18 (9)° with the benzene ring (C3–C8). Bond lengths (Allen *et al.*, 1987) and angles are within normal ranges.

In the crystal packing, Fig. 2, the molecules are linked *via* intermolecular O3—H1O3···O1^i^, O2—H1O2···O1^i^, N2—H1N2···O4^ii^ and N1—H1N1···O4^iii^ hydrogen bonds (Table 1) into one-dimensional chains along the [010] direction.

### Experimental

A mixture of ninhydrin (1.78 g) and urea (0.60 g) in molar ratio 1:1 were well dissolved in acetic acid and then heated over a water bath for 15 minutes. The reaction mixture was dried on rotavapor at low pressure to give the solid product which was then crystallized with alcohol-chloroform (1:1 *v/v*) mixture to give the colourless crystals of title compound (yield 100%, *m.p.* 490-493 K). IR (KBr): ν_max_ 3556, 3500 (N-H), 3312 (OH), 3175, 1727, 1682, 1605, 1429, 1340, 1296, 1218, 1179, 1113, 933, 742, 659. IR spectrum was taken on Shimadzu IR-408 Perkin Elmer 1800 (FTIR). The melting point was taken on Thermo Fisher digital melting point apparatus of IA9000 series and is uncorrected.

### Refinement

All H atoms were located in a difference Fourier map and refined freely [O—H = 0.89 (3)–0.94 (3) Å, N—H = 0.83 (2)–0.86 (2) Å, C—H = 0.96 (2)–1.01 (2) Å]. The highest residual electron density peak is located at 0.77 Å from C9 and the deepest hole is located at 0.68 Å from C1.

### Crystal data

**Table:**

C_10_H_8_N_2_O_4_	*Z* = 2
*M**_r_* = 220.18	*F*(000) = 228
Triclinic, *P*1	*D*_x_ = 1.598 Mg m^−^^3^
Hall symbol: -P 1	Mo *K*α radiation, λ = 0.71073 Å
*a* = 6.7914 (2) Å	Cell parameters from 2749 reflections
*b* = 7.3201 (2) Å	θ = 3.1–27.6°
*c* = 9.5006 (3) Å	µ = 0.13 mm^−^^1^
α = 94.258 (1)°	*T* = 296 K
β = 102.773 (1)°	Plate, colourless
γ = 93.725 (1)°	0.39 × 0.15 × 0.05 mm
*V* = 457.74 (2) Å^3^	

### Data collection

**Table:**

Bruker SMART APEXII CCD area-detector diffractometer	2081 independent reflections
Radiation source: fine-focus sealed tube	1634 reflections with *I* > 2σ(*I*)
graphite	*R*_int_ = 0.022
φ and ω scans	θ_max_ = 27.5°, θ_min_ = 2.2°
Absorption correction: multi-scan (*SADABS*; Bruker, 2009)	*h* = −8→8
*T*_min_ = 0.952, *T*_max_ = 0.994	*k* = −9→9
6684 measured reflections	*l* = −12→12

### Refinement

**Table:**

Refinement on *F*^2^	Primary atom site location: structure-invariant direct methods
Least-squares matrix: full	Secondary atom site location: difference Fourier map
*R*[*F*^2^ > 2σ(*F*^2^)] = 0.040	Hydrogen site location: inferred from neighbouring sites
*wR*(*F*^2^) = 0.110	H atoms treated by a mixture of independent and constrained refinement
*S* = 1.05	*w* = 1/[σ^2^(*F*_o_^2^) + (0.0511*P*)^2^ + 0.1396*P*] where *P* = (*F*_o_^2^ + 2*F*_c_^2^)/3
2081 reflections	(Δ/σ)_max_ = 0.001
177 parameters	Δρ_max_ = 0.25 e Å^−^^3^
0 restraints	Δρ_min_ = −0.23 e Å^−^^3^

### Special details

**Table:**

Experimental. The crystal was placed in the cold stream of an Oxford Cryosystems Cobra open-flow nitrogen cryostat (Cosier & Glazer, 1986) operating at 100.0 (1) K.
Geometry. All esds (except the esd in the dihedral angle between two l.s. planes) are estimated using the full covariance matrix. The cell esds are taken into account individually in the estimation of esds in distances, angles and torsion angles; correlations between esds in cell parameters are only used when they are defined by crystal symmetry. An approximate (isotropic) treatment of cell esds is used for estimating esds involving l.s. planes.
Refinement. Refinement of F^2^ against ALL reflections. The weighted R-factor wR and goodness of fit S are based on F^2^, conventional R-factors R are based on F, with F set to zero for negative F^2^. The threshold expression of F^2^ > 2sigma(F^2^) is used only for calculating R-factors(gt) etc. and is not relevant to the choice of reflections for refinement. R-factors based on F^2^ are statistically about twice as large as those based on F, and R- factors based on ALL data will be even larger.

### Fractional atomic coordinates and isotropic or equivalent isotropic displacement parameters (Å^2^)

**Table:**

	*x*	*y*	*z*	*U*_iso_*/*U*_eq_	
O1	1.26218 (17)	0.50614 (16)	0.93570 (12)	0.0382 (3)	
O2	0.73914 (19)	0.13075 (18)	0.94567 (13)	0.0394 (3)	
O3	0.59082 (18)	0.39814 (17)	0.75936 (14)	0.0376 (3)	
O4	0.8536 (2)	−0.15340 (16)	0.77260 (14)	0.0464 (4)	
N1	0.9431 (2)	0.46753 (19)	0.78463 (15)	0.0345 (3)	
N2	1.0569 (2)	0.23428 (18)	0.90164 (15)	0.0323 (3)	
C1	1.1014 (2)	0.4122 (2)	0.87930 (16)	0.0294 (3)	
C2	0.7722 (2)	0.3301 (2)	0.74365 (16)	0.0284 (3)	
C3	0.7435 (2)	0.2416 (2)	0.59059 (16)	0.0279 (3)	
C4	0.6895 (3)	0.3254 (3)	0.46259 (18)	0.0371 (4)	
C5	0.6634 (3)	0.2169 (3)	0.33296 (19)	0.0431 (5)	
C6	0.6904 (3)	0.0303 (3)	0.32948 (19)	0.0424 (4)	
C7	0.7470 (3)	−0.0527 (2)	0.45616 (18)	0.0358 (4)	
C8	0.7735 (2)	0.0553 (2)	0.58705 (17)	0.0290 (3)	
C9	0.8272 (2)	−0.0003 (2)	0.73466 (17)	0.0303 (4)	
C10	0.8466 (2)	0.1716 (2)	0.84121 (16)	0.0286 (3)	
H4A	0.672 (3)	0.461 (3)	0.467 (2)	0.048 (5)*	
H5A	0.617 (3)	0.274 (3)	0.245 (2)	0.056 (6)*	
H6A	0.670 (3)	−0.043 (3)	0.237 (2)	0.059 (6)*	
H7A	0.764 (3)	−0.183 (3)	0.455 (2)	0.048 (6)*	
H1O3	0.605 (4)	0.440 (3)	0.852 (3)	0.070 (7)*	
H1O2	0.735 (4)	0.242 (4)	1.002 (3)	0.089 (9)*	
H1N2	1.128 (3)	0.188 (3)	0.970 (2)	0.041 (5)*	
H1N1	0.935 (3)	0.580 (3)	0.765 (2)	0.049 (6)*	

### Atomic displacement parameters (Å^2^)

**Table:**

	*U*^11^	*U*^22^	*U*^33^	*U*^12^	*U*^13^	*U*^23^
O1	0.0395 (7)	0.0379 (7)	0.0335 (6)	−0.0069 (5)	0.0049 (5)	−0.0023 (5)
O2	0.0501 (8)	0.0369 (7)	0.0315 (6)	−0.0046 (5)	0.0115 (5)	0.0064 (5)
O3	0.0399 (7)	0.0384 (7)	0.0335 (7)	0.0137 (5)	0.0045 (5)	−0.0005 (5)
O4	0.0613 (9)	0.0239 (6)	0.0474 (8)	0.0061 (5)	−0.0038 (6)	0.0071 (5)
N1	0.0422 (8)	0.0218 (7)	0.0354 (8)	0.0001 (6)	−0.0006 (6)	0.0058 (6)
N2	0.0358 (8)	0.0279 (7)	0.0283 (7)	0.0019 (6)	−0.0040 (6)	0.0056 (5)
C1	0.0363 (8)	0.0285 (8)	0.0225 (7)	0.0006 (6)	0.0066 (6)	−0.0014 (6)
C2	0.0340 (8)	0.0225 (7)	0.0272 (8)	0.0045 (6)	0.0028 (6)	0.0037 (6)
C3	0.0291 (8)	0.0284 (8)	0.0257 (7)	0.0028 (6)	0.0040 (6)	0.0045 (6)
C4	0.0429 (10)	0.0378 (9)	0.0313 (9)	0.0058 (7)	0.0063 (7)	0.0108 (7)
C5	0.0442 (10)	0.0599 (12)	0.0259 (9)	0.0052 (9)	0.0068 (7)	0.0112 (8)
C6	0.0365 (10)	0.0602 (12)	0.0286 (9)	0.0014 (8)	0.0073 (7)	−0.0056 (8)
C7	0.0327 (9)	0.0353 (9)	0.0367 (9)	0.0032 (7)	0.0056 (7)	−0.0060 (7)
C8	0.0283 (8)	0.0277 (8)	0.0290 (8)	0.0018 (6)	0.0027 (6)	0.0021 (6)
C9	0.0317 (8)	0.0230 (7)	0.0329 (8)	0.0018 (6)	−0.0001 (6)	0.0036 (6)
C10	0.0341 (8)	0.0246 (7)	0.0250 (7)	0.0021 (6)	0.0015 (6)	0.0050 (6)

### Geometric parameters (Å, °)

**Table:**

O1—C1	1.2415 (18)	C2—C10	1.578 (2)
O2—C10	1.3939 (19)	C3—C4	1.390 (2)
O2—H1O2	0.94 (3)	C3—C8	1.391 (2)
O3—C2	1.392 (2)	C4—C5	1.385 (3)
O3—H1O3	0.89 (3)	C4—H4A	1.01 (2)
O4—C9	1.2145 (18)	C5—C6	1.388 (3)
N1—C1	1.346 (2)	C5—H5A	0.96 (2)
N1—C2	1.4496 (19)	C6—C7	1.378 (3)
N1—H1N1	0.86 (2)	C6—H6A	0.97 (2)
N2—C1	1.360 (2)	C7—C8	1.393 (2)
N2—C10	1.447 (2)	C7—H7A	0.96 (2)
N2—H1N2	0.83 (2)	C8—C9	1.464 (2)
C2—C3	1.513 (2)	C9—C10	1.535 (2)
			
C10—O2—H1O2	107.1 (16)	C3—C4—H4A	119.6 (11)
C2—O3—H1O3	108.0 (16)	C4—C5—C6	121.62 (16)
C1—N1—C2	113.31 (13)	C4—C5—H5A	117.3 (13)
C1—N1—H1N1	122.8 (14)	C6—C5—H5A	120.9 (13)
C2—N1—H1N1	122.7 (14)	C7—C6—C5	120.68 (17)
C1—N2—C10	112.65 (13)	C7—C6—H6A	119.2 (13)
C1—N2—H1N2	119.6 (13)	C5—C6—H6A	120.1 (13)
C10—N2—H1N2	122.8 (13)	C6—C7—C8	118.12 (17)
O1—C1—N1	126.05 (15)	C6—C7—H7A	121.2 (12)
O1—C1—N2	125.48 (15)	C8—C7—H7A	120.6 (12)
N1—C1—N2	108.47 (14)	C3—C8—C7	121.22 (15)
O3—C2—N1	112.93 (13)	C3—C8—C9	110.13 (14)
O3—C2—C3	108.64 (12)	C7—C8—C9	128.63 (15)
N1—C2—C3	113.22 (13)	O4—C9—C8	128.27 (15)
O3—C2—C10	115.83 (12)	O4—C9—C10	123.41 (14)
N1—C2—C10	102.17 (12)	C8—C9—C10	108.32 (12)
C3—C2—C10	103.71 (12)	O2—C10—N2	113.44 (12)
C4—C3—C8	120.46 (15)	O2—C10—C9	107.94 (12)
C4—C3—C2	127.23 (15)	N2—C10—C9	111.16 (13)
C8—C3—C2	112.30 (13)	O2—C10—C2	116.95 (13)
C5—C4—C3	117.90 (17)	N2—C10—C2	102.00 (11)
C5—C4—H4A	122.5 (11)	C9—C10—C2	104.99 (12)
			
C2—N1—C1—O1	−176.86 (15)	C6—C7—C8—C9	178.21 (15)
C2—N1—C1—N2	3.96 (19)	C3—C8—C9—O4	175.78 (16)
C10—N2—C1—O1	170.05 (15)	C7—C8—C9—O4	−2.4 (3)
C10—N2—C1—N1	−10.77 (19)	C3—C8—C9—C10	−4.17 (17)
C1—N1—C2—O3	128.62 (15)	C7—C8—C9—C10	177.62 (15)
C1—N1—C2—C3	−107.37 (15)	C1—N2—C10—O2	−114.40 (15)
C1—N1—C2—C10	3.51 (18)	C1—N2—C10—C9	123.74 (14)
O3—C2—C3—C4	60.0 (2)	C1—N2—C10—C2	12.28 (17)
N1—C2—C3—C4	−66.3 (2)	O4—C9—C10—O2	−47.5 (2)
C10—C2—C3—C4	−176.23 (15)	C8—C9—C10—O2	132.48 (13)
O3—C2—C3—C8	−118.71 (14)	O4—C9—C10—N2	77.5 (2)
N1—C2—C3—C8	114.97 (15)	C8—C9—C10—N2	−102.51 (14)
C10—C2—C3—C8	5.04 (17)	O4—C9—C10—C2	−172.92 (15)
C8—C3—C4—C5	1.1 (2)	C8—C9—C10—C2	7.03 (16)
C2—C3—C4—C5	−177.49 (16)	O3—C2—C10—O2	−7.76 (19)
C3—C4—C5—C6	−0.2 (3)	N1—C2—C10—O2	115.42 (14)
C4—C5—C6—C7	−0.8 (3)	C3—C2—C10—O2	−126.69 (13)
C5—C6—C7—C8	0.8 (3)	O3—C2—C10—N2	−132.12 (13)
C4—C3—C8—C7	−1.2 (2)	N1—C2—C10—N2	−8.94 (15)
C2—C3—C8—C7	177.66 (14)	C3—C2—C10—N2	108.95 (13)
C4—C3—C8—C9	−179.53 (14)	O3—C2—C10—C9	111.83 (14)
C2—C3—C8—C9	−0.71 (18)	N1—C2—C10—C9	−124.99 (13)
C6—C7—C8—C3	0.2 (2)	C3—C2—C10—C9	−7.09 (15)

### Hydrogen-bond geometry (Å, °)

**Table:**

*D*—H···*A*	*D*—H	H···*A*	*D*···*A*	*D*—H···*A*
O3—H1O3···O1^i^	0.89 (3)	2.02 (3)	2.8653 (17)	158 (2)
O2—H1O2···O1^i^	0.95 (3)	1.89 (3)	2.8103 (17)	163 (2)
N2—H1N2···O4^ii^	0.83 (2)	2.454 (19)	3.1282 (19)	139.3 (18)
N1—H1N1···O4^iii^	0.86 (2)	2.06 (2)	2.8841 (18)	159.4 (19)

Symmetry codes: (i) −*x*+2, −*y*+1, −*z*+2; (ii) −*x*+2, −*y*, −*z*+2; (iii) *x*, *y*+1, *z*.
